# Chemotherapy in Combination With Immune Checkpoint Inhibitors for the First-Line Treatment of Patients With Advanced Non-small Cell Lung Cancer: A Systematic Review and Literature-Based Meta-Analysis

**DOI:** 10.3389/fonc.2019.00264

**Published:** 2019-04-16

**Authors:** Alfredo Addeo, Giuseppe Luigi Banna, Giulio Metro, Massimo Di Maio

**Affiliations:** ^1^Department of Oncology, University Hospital of Geneva, Geneva, Switzerland; ^2^Division of Medical Oncology, Cannizzaro Hospital, Catania, Italy; ^3^Medical Oncology, Santa Maria della Misericordia Hospital, Azienda Ospedaliera di Perugia, Perugia, Italy; ^4^Department of Oncology, Ordine Mauriziano Hospital, University of Turin, Turin, Italy

**Keywords:** NCSLC, checkpoint inhibition, first line, PDL1, PD1

## Abstract

**Background:** Checkpoint inhibitors plus platinum-based chemotherapy have shown superiority compared to chemotherapy alone as first-line therapy in advanced non–small cell lung carcinoma (NSCLC). To evaluate the relative benefit in term of Overall Survival (OS) and Progression-free Survival (PFS) of checkpoint inhibitors plus chemotherapy vs. chemotherapy alone, overall and in subgroups defined by PDL1 expression we have performed a meta-analysis.

**Data Sources:** This meta-analysis searched PubMed and checked references of the selected English language articles to identify further eligible trials. Data collection for this study took place from October 1 to October 24, 2018.

**Results:** In total, 8 trials involving 4,646 patients with advanced NSCLC, 3.314 (71%) and 1.332 (29%) with a non-squamous and squamous histology, respectively, were included in this meta-analysis. Four trials used atezolizumab, 3 pembrolizumab, and 1 nivolumab, accounting for 2.985 (64%), 1.298 (28%), and 363 (8%) of patients, respectively. The patients were randomized to receive first-line chemotherapy plus a checkpoint inhibitor vs. first-line chemotherapy, 2,978 patients for the OS endpoint and first-line chemotherapy plus a checkpoint inhibitor vs. first-line chemotherapy, 1,740 patients in the PFS endpoint. Checkpoint inhibitors plus chemotherapy were associated with prolonged OS, compared with chemotherapy in the ITT population (HR, 0.74; 95% CI, 0.64–0.87; *p* = 0.0002, with significant heterogeneity among trials). Notably within the PDL1 low group (1–49) there was a significant heterogeneity (*p* = 0.06) between type of drug and efficacy: the combination of chemotherapy plus pembrolizumab showed an OS benefit (HR, 0.56; 95% CI, 0.40–0.78; *P* < 0.00007) unlike the atezolizumab backbone trials (HR, 0.92; 95% CI, 0.62–1.37; *P* < 0.69). However, checkpoint inhibitors plus chemotherapy were associated with prolonged PFS in the ITT (HR, 0.61; 95% CI, 0.56–0.66; *P* < 0.00001) and across PDL1 subgroups.

**Conclusion and Relevance:** Checkpoint inhibitors plus chemotherapy compared with chemotherapy, are associated with significantly prolonged OS and PFS in first-line therapy in NSCLC. In the low PDL1 subgroups the benefit was statistically significant only in the pembrolizumab backbone trials. The findings of this meta-analysis could assist in the design and interpretation of future trials and in economic analyses.

## Introduction

Lung cancer is the leading cause of cancer death worldwide. Non-small-cell lung cancer (NSCLC) accounts for almost 85% of all cases (1). The prognosis of NSCLC patients remains quite unsatisfactory despite significant progress in the past few years (2, 3). Inhibitors of programmed death-1 (PD-1) and its ligand PD-L1 have proven to be effective therapies in metastatic NSCLC lacking sensitizing *EGFR* or *ALK* mutations, initially as second-line therapy (4–8). Subsequently, in metastatic NSCLC patients with PD-L1 expression of at least 50% on tumor cells, upfront pembrolizumab improved median progression-free survival (PFS) and overall survival (OS) compared to standard platinum-based chemotherapy (9). However, patients with a tumor proportion score (TPS) of 50% or greater represent only 20–30% of those with NSCLC (10). To enhance the immune response through PD-1 inhibition, several studies have combined the potential immunogenic effects of cytotoxic chemotherapy with immune checkpoint inhibitors (ICPI). The first study that gave some important information regarding the efficacy of combining IO and chemotherapy (CH) was Keynote 021 (11): a randomized phase II trial of carboplatin plus pemetrexed with and without pembrolizumab. It showed significantly better response rates (RR) and longer PFS with the addition of pembrolizumab to chemotherapy. Several subsequent studies have been published and presented at international conferences, showing a benefit in terms of PFS and/or OS in the intention-to-treat (ITT) population. However, due to the trials' design, where subgroup analysis by PD-L1 breakdown was mainly exploratory, the question about the magnitude of benefit in the three main different subgroups (PD-L1 negative, low or high) has remained rather uncertain.

We have therefore conducted a meta-analysis to compare the PFS and OS of chemotherapy plus ICPI vs. chemotherapy in the ITT population and within the three principal subgroups of PD-L1 expression (negative, low or high).

## Materials and Methods

### Evidence Acquisition

#### Identification of Eligible Trials

A PubMed literature search was performed in October 2018 and updated the 24th of October 2018, to identify all randomized trials testing the addition of an antiPD-1 or antiPD-L1 ICPI to first-line platinum-based chemotherapy in patients with NSCLC. The following key-words were used: (non-small cell lung cancer) AND nivolumab OR pembrolizumab OR atezolizumab OR avelumab OR durvalumab) AND (random^*^). References of the selected articles were also checked to identify other eligible trials. Furthermore, proceedings of the main international meetings (American Society of Clinical Oncology [ASCO] annual meeting, European Society of Medical Oncology [ESMO] annual meeting, International Association for the Study of Lung Cancer [IASLC] World Conference on Lung Cancer), were searched from 2010 onwards for relevant abstracts. Both trials enrolling patients with tumor histology (squamous and non-squamous) and trials enrolling only patients with one type of histology were eligible. Trials with treatment arms including a targeted agent (e.g., bevacizumab) in addition to platinum-based chemotherapy and trials with a maintenance phase (e.g., pemetrexed) after the completion of platinum-based chemotherapy were considered eligible for the analysis. When more than one report was available for the same clinical trial, the most recent information (corresponding to longer follow-up and higher number of events) was considered in the analysis.

### Data Collection

We have collected aggregate data from publications or presentations at meetings and for each eligible trial, the following data were extracted, if available:

- main inclusion criteria: age, performance status, stage, histology;
- details of study treatment: type of platinum-based chemotherapy (drugs, doses, and number of cycles), type of ICPI (drug, dose, and duration of treatment);
- study design: primary endpoint, study hypothesis;
- patient enrolment and follow-up: accrual start and end date; number of patients assigned to the experimental arm (chemotherapy + ICPI), number of patients assigned to the control arm (chemotherapy alone), median follow-up;
- OS: number of deaths in each arm, median OS, hazard ratio with 95% confidence interval, *p*-value, details of subgroup analysis according to PD-L1 expression (negative expression; low expression; high expression);
- PFS: number of events in each arm, median PFS, hazard ratio with 95% confidence interval, *p*-value, details of subgroup analysis according to PD-L1 expression (negative expression; low expression; high expression).

Low PD-L1 expression was defined as PD-L1 TPS of 1–49% in the trials with pembrolizumab or PD-L1 expression on 1–49% of tumor cells (TC) or 1–9% of tumor-infiltrating immune cells (IC) in the trials with atezolizumab. High PD-L1 expression was defined as PD-L1 TPS of 50% or greater in the trials with pembrolizumab or PD-L1 expression of 50% or greater on TC or 10% or greater IC in the trials with atezolizumab.

### Statistical Methods

After data were abstracted, analysis was performed with the Review Manager (RevMan 5.3) software. In all the 8 trials included (11–20), efficacy data were analyzed from all randomly assigned patients on an ITT basis. The primary endpoint of the meta-analysis was OS. The secondary endpoint was PFS.

For both OS and PFS, the summary measure was the hazard ratio (with 95% confidence interval). A random-effects model was applied. Statistical heterogeneity among studies was examined using the χ ^2^ test and the *I*^2^ statistic, which expresses the percentage of the total observed variability due to study heterogeneity.

One trial (16, 17) had two experimental arms adding an ICPI to chemotherapy, the first testing the combination of carboplatin + paclitaxel, bevacizumab and atezolizumab, and the second testing the combination of carboplatin + paclitaxel and atezolizumab, vs. the same control arm without ICPI (carboplatin + paclitaxel + bevacizumab). We decided to include both comparisons in the meta-analysis. However, since that trial used the same control arm for the two comparisons, the weight of each comparison was reduced according to a correction factor: the standard error for each comparison was multiplied by the square root of (2+1)/2) = 1.225. This correction resulted in a prudential increase in the width of the confidence interval for the estimated hazard ratio of each comparison.

The subgroup analysis of patients according to PD-L1 expression was available for 5 trials (12, 14–16, 19) for OS and 8 trials (11, 12, 14–16, 18–20) for PFS. In the KEYNOTE-021 trial (11), information about subgroup analysis of PFS was available for patients with absent PD-L1 expression and for patients with high PD-L1 expression, but not for patients with low PD-L1 expression. In the CheckMate 227 trial (20) the comparison between chemotherapy + immune checkpoint inhibitor vs. chemotherapy alone was conducted only in the subgroup of cases with no PD-L1 expression, so this trial was considered only in this subgroup analysis.

For both OS and PFS, in the whole population and in the subgroup analysis according to PD-L1 expression, the heterogeneity among the subsets of trials with different immune checkpoint inhibitors was assessed using an interaction test. The null hypothesis that the efficacy of the addition of immune checkpoint inhibitor to chemotherapy is equal with different drugs was tested with a χ^2^ test.

### Role of Funding Source

There was no funding source for this systematic review and meta-analysis. All authors had full access to all the data and the corresponding author (Alfredo Addeo) had final responsibility for the decision to submit for publication.

## Results

### Characteristics and Quality of the Trials

The selection process of trials eligible for the meta-analysis is reported in Supplemental Figure 1. In the search updated on October 24th 2018, out of the 273 papers published *in extenso*, 270 were excluded, while three were found eligible for inclusion (11, 12, 16). Five further eligible trials were found searching the proceedings of the main international meetings (14, 15, 17–20).

The main characteristics of the eight available trials are described in Table 1. Considering all the selected trials, 4,646 patients were included, 3,314 (71%) and 1,332 (29%) with a non-squamous and squamous histology, respectively. Five trials were with atezolizumab, 3 were with pembrolizumab and 1 with nivolumab, accounting for 2,985 (64%), 1,298 (28%), and 363 (8%) patients, respectively. Chemotherapy regimens included platinum-pemetrexed for 1,590 patients (34%) with non-squamous histology, carboplatin-(nab)-paclitaxel (with the possible addition of bevacizumab in non-squamous) for 2,966 patients (64%) with both histology, and platinum-gemcitabine for 90 patients (2%) with squamous histology.

**Table 1:** Main characteristics of the selected studies.

**Trialreference**	**Drug**	**Phase no. pts**	**Histology**	**PD-L1**	**FU time median mo**.	**HR OS (95% CI) *P*-value**	**HR PFS (95% CI) *P*-value**
KN-189 (1)	Pembrolizumab ± Platinum-Pem	III 616	NonSq	any	10.5	0.49 (0.38–0.64) <0.001	0.52 (0.43–0.64) <0.001
KN-021 (2, 3)	Pembrolizumab ± Carbo-Pem	II 123	NonSq	any	10.6	0.90 (0.42–1.91) 0.39	0.53 (0.31–0.91) 0.010
							
KN-407^a^ (4)	Pembrolizumab ± Carbo-(nab)Pac	III 559	Sq	any	7.7	0.64 (0.49–0.85) 0.0008	0.56 (0.45–0.70) <0.0001
IMPower131 (5)^a^	Atezolizumab ± Carbo-nabPac	III 683	Sq	any	9.8^b^	0.96 (0.78–1.18) 0.69	0.71 (0.60–0.85) 0.0001
IMPower150 (6)	Atezolizumab ± Carbo-Pac-Beva	III 696	NonSq	any	15.5	0.78^a^ (0.64–0.96) *P* = 0.02	0.62 (0.52–0.74) *P* <0.001
IMPower150bis (7)	Atezolizumab + Carbo-Pac vs. Carbo-Pac-Beva	III 686^c^	NonSq	any	20.0	NR	0.88 ^a^ (0.72-1.08) 0.20
IMPower132 (8)^a^	Atezolizumab ± Platinum-Pem	III 578	NonSq	any	NR	0.81 (0.64–1.03) *p* = 0.08	0.60 (0.49–0.72) *P* <0.0001
IMPower130 (9)	Atezolizumab ± Carbo-nabPac	III 679	NonSq	any	13.0^b^	0.79 (0.64–0.98) 0.03	0.64 (0.54–0.77) <0.0001
CM-227 (10)	Nivolumab ± Platinum-Pem in NonSq Platinum-Gem in Sq	III 363	NonSq (273) Sq (90)	<1%	11.2^b^	NR	0.74 (0.58–0.94) *P* = NR

*Beva, bevacizumab; Carbo, carboplatin; CM, Checkmate; ECOG PS, Eastern Cooperative Oncology Group Performance Status; ICPIs, immune-checkpoint inhibitors; CTRT, chemo-radiotherapy; FU, follow-up; KN, Keynote; mo., months; NonSq, non-squamous; NR, not reported; NSCLC, non-small cell lung cancer; Pem, pemetrexed; Pac, paclitaxel; Sq, squamous; vs. versus*.

a *Results refer to first interim analysis*.

b *Minimum follow-up*.

c *Carbo-Pac-Beva arm was the same of the above mentioned trial and included 337 patients*.

### Patient Characteristics

**Figure 1:**
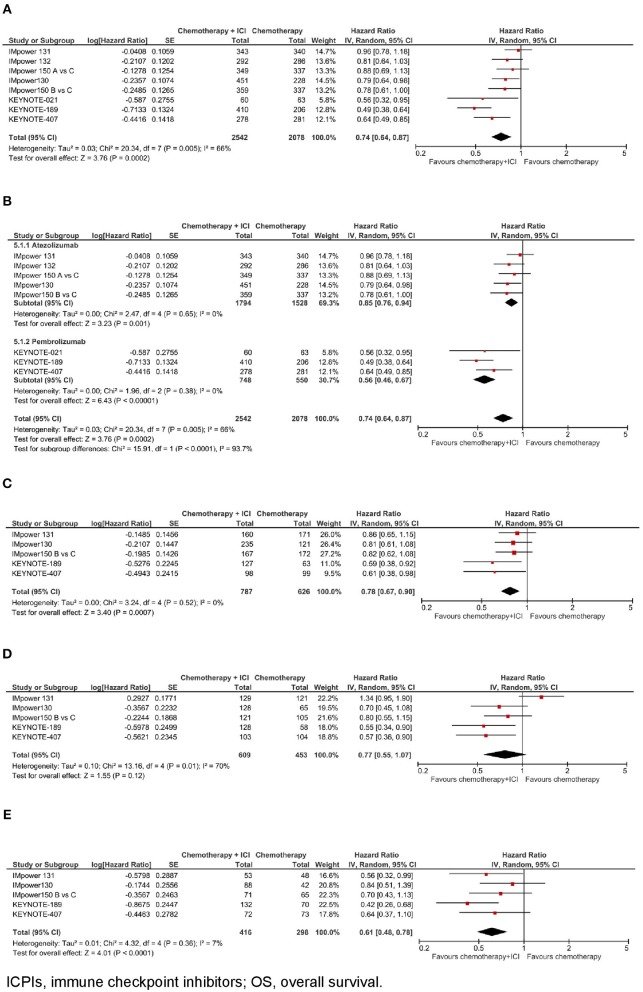
Overall survival with chemotherapy plus ICPIs. **(A)** OS in whole study population. **(B)** OS by ICPI administered. **(C)** OS by PD-L1 expression- PD-L1 negative. **(D)** OS by PD-L1 expression- PD-L1 low. **(E)** OS by PD-L1 expression- PD-L1 high.

**Figure 2:**
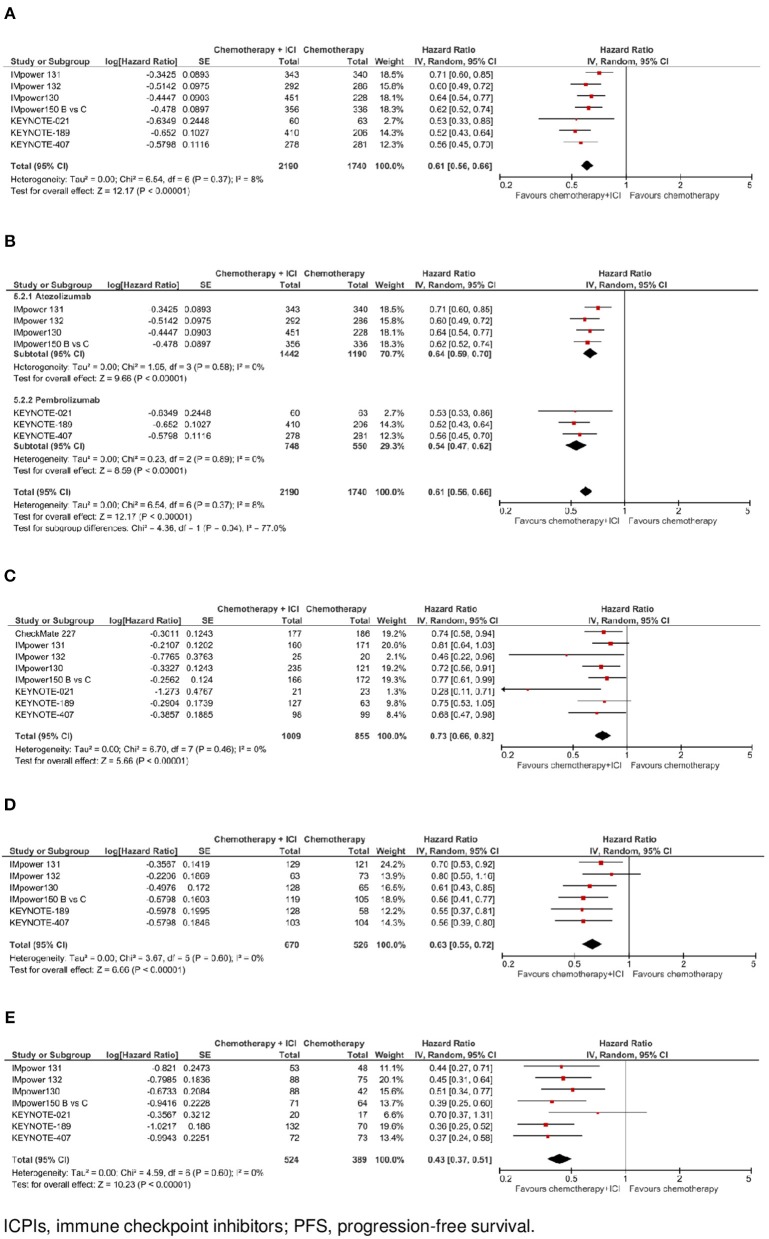
Progression-free survival with chemotherapy plus ICPI. **(A)** PFS in whole study population. **(B)** PFS by ICPI administered. **(C)** PFS by PD-L1 expression- PD-L1 negative. **(D)** PFS by PD-L1 expression - PD-L1 low. **(E)** PFS by PD-L1 expression- PD-L1 high.

Overall, 4,620 patients were enrolled in the 8 trials included in the meta-analysis (OS comparison in the whole population), 2,542 (55.0%) assigned to platinum-based chemotherapy + ICPI, and 2,078 (45.0%) assigned to platinum-based chemotherapy alone (Table 1 and Figure 1A). In addition, 363 patients enrolled in the CheckMate 227 trial (20) were considered only for the PFS comparison in the subgroup of cases with negative PD-L1 expression (Table 1 and Figure 2C). Main characteristics of the enrolled patients are described in Table 2. For 4 trials the enrolment period was available, and patients were enrolled between November 2014 and March 2017. Median age was 62.5–65 years and all patients had a 0 or 1 Eastern Cooperative Oncology Group (ECOG) performance status (PS), with the proportion of patients with PS 0 and 1 ranging from 31 to 60% and 40 to 64%, respectively. Information about PD-L1 expression was available for 3,808 of the 3,862 evaluable patients (99%). With the exception of the CheckMate 227 study (20), which was restricted to those with negative PD-L1 expression, the proportion of patients with negative PD-L1 expression ranged from 31 to 37% and 47 to 53% for patients enrolled in trials with pembrolizumab or atezolizumab, respectively. The proportion of patients with low PD-L1 expression ranged from 28 to 37% and 28 to 38% in studies with pembrolizumab and atezolizumab, respectively. The proportion of patients with high PD-L1 expression ranged from 26 to 34% and 14 to 20% for trials with pembrolizumab and atezolizumab, respectively.

**Table 2:** Main characteristics of enrolled patients.

**Trialreference**	**Arm (accrual period)**	**No. pts**	**Age, yr, median**	**ECOG PS 0, 1, 2 (%)**	**PD-L1 NE (%)**	**PD-L1^**a**^ neg, low, high (%)**
KN-189 (1)	Pembro-Combo vs. Placebo-Combo (02/2016-03/2017)	410 206	65 63.5	45, 54, 0.2 39, 61, 0	6 7	31, 31, 32 31, 28, 34
KN-021 (2, 11)	Pembro-Chemo vs. Chemo (11/2014-01/2016)	60 63	62.5 63	40, 58, 0 46, 54, 0	0 0	35, 32, 33 37, 37, 27
KN-407^4^	Pembro-Chemo vs. Placebo-Chemo (NR)	278 281	65 65	26, 74, 0 32, 68, 0	2.5 2	34, 37, 26 35, 37, 26
IMPower131 (5)	Atezo-Chemo vs. Chemo (NR)	343 340	65 65	34, 66, 0 32, 68, 0	0.3 0	47, 38, 15 50, 36, 14
IMPower150 (6)	Atezo-Chemo-Beva vs. Chemo-Beva (03/2015-12/2016)	400 400	63 63	40, 60, 0 45, 55, 0	0 0	48, 33, 19 51, 31, 18
IMPower150bis (7)	Atezo-Chemo vs. Chemo-Beva (03/2015-12/2016)	402 400	63 63	45, 55, 0 45, 55, 0	0.2 0	47, 36, 17 51, 31, 18
IMPower132 (8)	Atezo-Chemo Placebo-Chemo (NR)	292 286	64 63	43, 57, 0 40, 60,0	NR NR	NR NR
IMPower130 (9)	Atezo-Chemo Placebo-Chemo (NR)	451 228	NR NR	58, 42, 0 60, 40, 0	0 0	52, 28, 20 53, 29, 18
CM-227 (10)^b^	Nivo-Chemo vs. Chemo (NR)	177 186	64 64	33, 66, NR 31, 68, NR	NA NA	100, 0, 0 100, 0, 0

*Atezo, atezolizumab; Beva, bevacizumab; Carbo, carboplatin; Chemo, chemotherapy; ECOG PS, Eastern Cooperative Oncology Group Performance Status; IC, tumor-infiltrating immune cell; ICPIs, immune-checkpoint inhibitors; NA, not applicable; NE, not evaluated; neg, negative; NR, not reported; Nivo, nivolumab; Pembro, pembrolizumab; vs, versus; TC, tumor cell*.

a *For Pembro, negative = <1%, low = 1-49%, high ≥ 50% by the use of the 22C3 pharmDx assay (Agilent); for Atezo, negative = TC0 and IC0; low = TC 1/2 or IC 1/2; high = TC3 or IC3 by the use of the SP142 PD-L1 immunohistochemistry assay (Ventana Medical Systems)*.

b *Study results restricted to patients with <1% PD-L1 expression*.

### Overall Survival

In the whole study population (*N* = 4.620), as shown in Figure 1A, the addition of an ICPI to platinum-based chemotherapy in patients with metastatic NSCLC was associated with a statistically significant benefit in overall survival (hazard ratio [HR] 0.74, 95% confidence interval [CI] 0.64–0.87, *p* = 0.0002). There was evidence of statistically significant heterogeneity among the 8 comparisons (*p* = 0.005, *I*^2^ = 66%). HR was equal to 0.85 (95% CI 0.76–0.94, *p* = 0.001) in the trials with atezolizumab, and equal to 0.56 (95% CI 0.46–0.67, *p* < 0.00001) in the trials with pembrolizumab, with statistically significant quantitative interaction between type of drug and treatment efficacy (interaction *p* < 0.0001; see Figure 1B).

**Figure 3:**
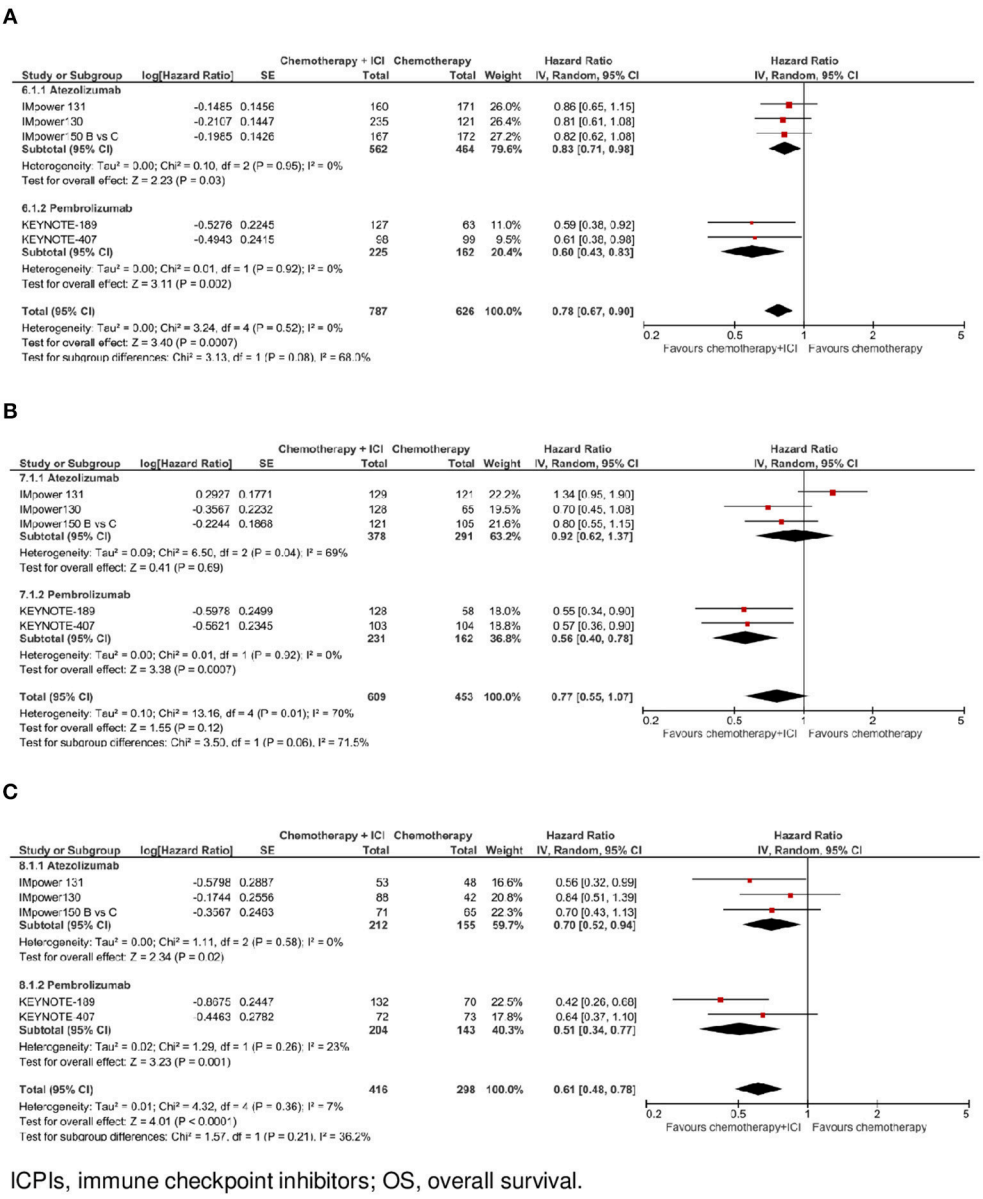
Overall survival by ICPI drug according to PD-L1 expression. **(A)** OS in PO-L1 negative population. **(B)** OS in PO-L1 low population. **(C)** OS in PO-L1 high population.

In the subgroup of patients with negative PD-L1 expression (*N* = 1.413, data available for 5 trials), as shown in Figure 1C, the addition of an ICPI to platinum-based chemotherapy in patients with metastatic NSCLC was associated with a statistically significant benefit in OS (HR 0.78, 95% CI 0.67–0.90, *p* = 0.0007). There was no evidence of statistically significant heterogeneity among the 5 trials (*p* = 0.52, *I*^2^ = 0%). HR was equal to 0.83 (95% CI 0.71–0.98, *p* = 0.03) in the 3 trials with atezolizumab, and equal to 0.60 (95% CI 0.43–0.83, *p* = 0.002) in the 2 trials with pembrolizumab, with evidence of a borderline statistically significant quantitative interaction between type of drug and treatment efficacy (interaction *p* = 0.08; see Figure 3A).

In the subgroup of patients with low PD-L1 expression (*N* = 1,062, data available for 5 trials), as shown in Figure 1D, the addition of an ICPI to platinum-based chemotherapy in patients with metastatic NSCLC was not associated with a statistically significant benefit in OS (HR 0.77, 95% CI 0.55–1.07, *p* = 0.12). There was evidence of statistically significant heterogeneity among the 5 trials (*p* = 0.01, *I*^2^ = 70%). HR was equal to 0.92 (95% CI 0.62–1.37, *p* = 0.69) in the 3 trials with atezolizumab, and equal to 0.56 (95% CI 0.40–0.78, *p* = 0.0007) in the 2 trials with pembrolizumab, with evidence of statistically significant quantitative interaction between type of drug and treatment efficacy (interaction *p* = 0.06; see Figure 3B).

In the subgroup of patients with high PD-L1 expression (*N* = 714, data available for 5 trials), as shown in Figure 1E, the addition of an ICPI to platinum-based chemotherapy in patients with metastatic NSCLC was associated with a statistically significant benefit in OS (HR 0.61, 95% CI 0.48–0.78, *p* < 0.0001). There was no evidence of statistically significant heterogeneity among the 5 trials (*p* = 0.36, *I*^2^ = 7%). HR was equal to 0.70 (95% CI 0.52–0.94, *p* = 0.02) in the 3 trials with atezolizumab, and equal to 0.51 (95% CI 0.34–0.77, *p* = 0.001) in the 2 trials with pembrolizumab, with no evidence of interaction between type of drug and treatment efficacy (interaction *p* = 0.21; see Figure 3C).

### Progression-Free Survival

In the whole study population (*N* = 3.930, data available for 7 trials), as shown in Figure 2A, the addition of an ICPI to platinum-based chemotherapy in patients with metastatic NSCLC was associated with a statistically significant benefit in PFS(HR 0.61, 95% CI 0.56–0.66, *p* < 0.00001). There was no evidence of statistically significant heterogeneity among the 7 trials (*p* = 0.37, *I*^2^ = 8%). HR was equal to 0.64 (95% CI 0.59–0.70, *p* < 0.00001) in the 4 trials with atezolizumab, and equal to 0.54 (95% CI 0.47–0.62, *p* < 0.00001) in the 3 trials with pembrolizumab, with evidence of quantitative interaction between type of drug and treatment efficacy (interaction *p* = 0.04; see Figure 2B).

**Figure 4:**
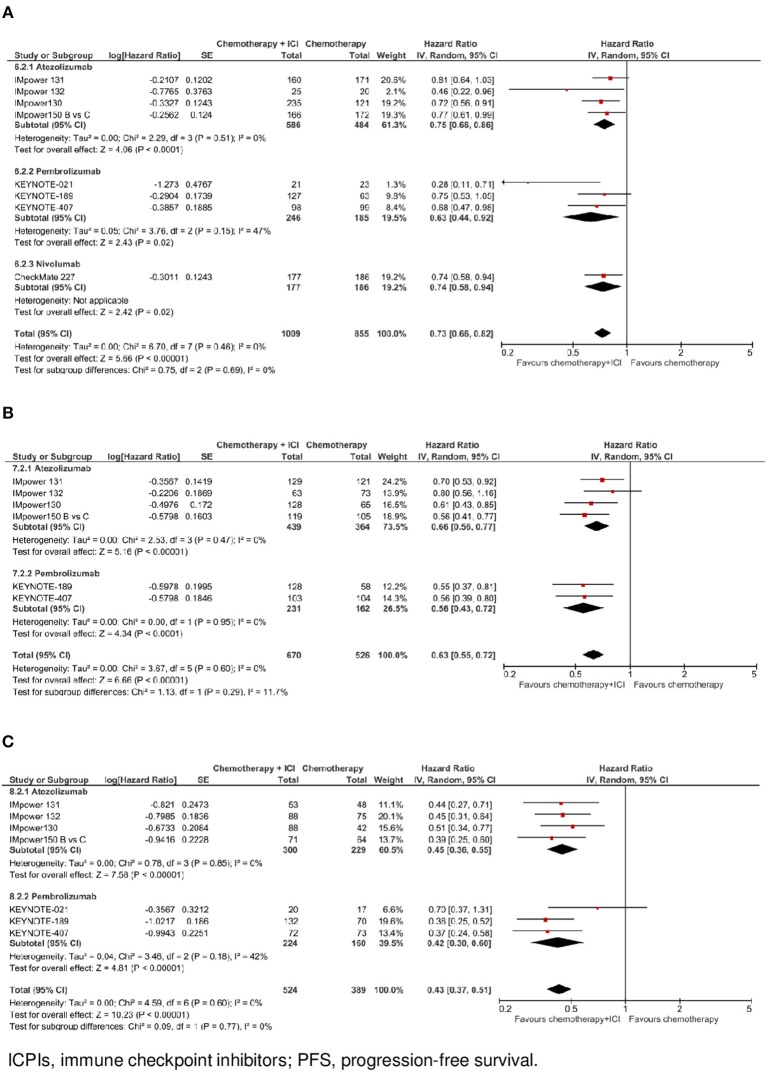
Progression-free survival by ICPI drug according to PD-L1 expression. **(A)** PFS in PO-L1 negative population. **(B)** PFS in PO-L1 low population. **(C)** PFS in PO-L1 high population.

In the subgroup of cases with negative PD-L1 expression (*N* = 1,864, data available for 8 trials), as shown in Figure 2C, the addition of an ICPI to platinum-based chemotherapy in patients with metastatic NSCLC was associated with a statistically significant benefit in PFS (HR 0.73, 95% CI 0.66–0.82, *p* < 0.00001). There was no evidence of statistically significant heterogeneity among the 8 trials (*p* = 0.46, *I*^2^ = 0%). HR was equal to 0.75 (95% CI 0.66–0.86, *p* < 0.0001) in the 4 trials with atezolizumab, equal to 0.63 (95% CI 0.44–0.92, *p* = 0.02) in the 3 trials with pembrolizumab, and equal to 0.74 (95% CI 0.58–0.94, *p* = 0.02) in the trial with nivolumab, without evidence of significant interaction between type of drug and treatment efficacy (interaction *p* = 0.69; see Figure 4A).

In the subgroup of patients with low PD-L1 expression (*N* = 1,196, data available for 6 trials), as shown in Figure 2D, the addition of an ICPI to platinum-based chemotherapy in patients with metastatic NSCLC was associated with a statistically significant benefit in PFS (HR 0.63, 95% CI 0.55–0.72, *p* < 0.00001). There was no evidence of statistically significant heterogeneity among the 6 trials (*p* = 0.60, *I*^2^ = 0%). HR was equal to 0.66 (95% CI 0.56–0.77, *p* < 0.00001) in the 4 trials with atezolizumab, and equal to 0.56 (95% CI 0.43–0.72, *p* < 0.0001) in the 2 trials with pembrolizumab, without evidence of significant interaction between type of drug and treatment efficacy (interaction *p* = 0.29; see Figure 4B).

In the subgroup of patients with high PD-L1 expression (*N* = 913, data available for 7 trials), as shown in Figure 2E, the addition of an ICPI to platinum-based chemotherapy in patients with metastatic NSCLC was associated with a statistically significant benefit in PFS (HR 0.43, 95% CI 0.37–0.51, *p* < 0.00001). There was no evidence of statistically significant heterogeneity among the 7 trials (*p* = 0.60, *I*^2^ = 0%). HR was equal to 0.45 (95% CI 0.36–0.55, *p* < 0.00001) in the 4 trials with atezolizumab and equal to 0.42 (95% CI 0.30–0.60, *p* < 0.00001) in the 3 trials with pembrolizumab, without evidence of significant interaction between type of drug and treatment efficacy (interaction *p* = 0.77; see Figure 4C).

## Discussion

In the present meta-analysis of all published and presented randomized clinical trials with PD-1 and PD-L1 inhibitors plus chemotherapy as first-line treatment for patients with metastatic NSCLC, we observed a clear benefit in OS and PFS in the ITT population with the addition of ICPI to chemotherapy.

We addressed the question regarding the benefit of chemotherapy plus ICPI in terms of OS and PFS in all different PD-L1 expression subgroups. Furthermore, to extend the analysis, we also explored the possible difference in terms of OS and PFS by grouping trials by anti-PD-1 or anti-PD-L1 drugs.

As shown in the results, there is a PFS advantage of ICPI plus chemotherapy over chemotherapy alone both in the ITT population and in the different subgroups according to PD-L1 expression and type of drug. However, while the OS benefit is found in the overall ITT population, it does not reach statistical significance in the ITT PD-L1 low expression population. Significant heterogeneity appears in the ITT ICPI subgroup analysis in favor of the anti-PD-1 ICPI pembrolizumab. Furthermore, along with the clear OS advantage observed with the addition of ICPIs to chemotherapy in the ITT population and in the PD-L1 high subgroup of patients, when examining the heterogeneity between different drugs the HRs are more beneficial with the anti-PD-1 pembrolizumab than with the anti-PD-L1 atezolizumab in the ITT and in the PD-L1 negative and low expression subgroup of patients. This evidence suggests the benefit in OS is, at least currently, strongly driven by the Keynote trials (11–14), with at least three possible explanations.

The first relies on the more mature data and longer follow-up of Keynote trials with pembrolizumab, of which two (11, 12) out of three (11–14) were already published *in extenso*, as compared with the Impower studies with atezolizumab, from which data are mainly preliminary. (15–19). Hence, data might change one way or another during the next 12–18 months, requiring confirmation with longer follow-up as soon as the final results are published.

The second aspect pertains to the reliability of the immunohistochemistry testing and scoring used in the Impower trials to identify and stratify patients according to their PD-L1 tumor expression. This aspect has been extensively assessed in the Blueprint phase 1 project (BP1) which clearly showed that three PD-L1 assays (22C3, 28-8, and SP263) had comparable analytical performance for assessment of PD-L1 expression on tumor cells (TCs), whereas the SP-142 PD-L1 assay appeared to stain fewer TCs compared with the other (21) assays. In contrast, all the assays stained tumor-infiltrating immune cells (ICs), but with poor concordance between assays. These findings were further confirmed in the Bluprint phase 2 project >(BP2) (22), which consolidates the analytical evidence for interchangeability of the 22C3, 28-8, and SP263 assays and lower sensitivity of the SP142 assay for determining tumor proportion score on TCs. Moreover, we have highlighted a clear difference in the proportion of patients in negative and high PD-L1 expression subgroups, and a heterogeneity in the low PD-L1 expression subgroup comparing pembrolizumab backbone trials and Atezolizumab backbone ones. This, once again, confirms the essential role of the platform used for testing PD-L1 and raises the question of whether a companion diagnostic should be preferred to reliably reproduce the benefits reported by clinical trials in clinical practice.

The third aspect, perhaps more provocative but at the same time fascinating, is the possibility that there was a real difference in efficacy between anti-PD-1 and anti-PD-L1 drugs, or at least between pembrolizumab and atezolizumab. Depending on results after a longer follow-up, this may warrant further exploration in a hypothetical randomized trial. There could also be differences in the immunogenic activities of the chemotherapy compounds combined with ICPI, as reported by Novosiadly et al. (23), suggesting differently an immunomodulatory effect of pemetrexed compared to paclitaxel. However, the magnitude of the effect we observed in our analysis with the Keynote 407 trial, which combined the anti-PD-1 pembrolizumab with paclitaxel or nab-paclitaxel in squamous histology, appears similar to other Keynote trial results.

Our study presents some limitations that we want to acknowledge. First of all, this meta-analysis relies on published results rather than on individual patients' data, thus the results from subgroup analysis are merely suggestive. Secondly, the OS data from the included trials were not mature enough, so the data might change in the future and, hence, updating the meta-analysis with final OS data will be essential. A third aspect is that only the Impower 150 recruited NSCLC patients with activating EGFR mutation or ALK rearrangement, and that used Bevacizumab as part of the backbone treatment. For both aspects it is difficult to quantify the impact on our final results analysis.

## Conclusions

The results of this meta-analysis suggest that the combination of chemotherapy plus ICPI, irrespective of histology and PD-L1 expression, should be considered as the new standard for patients with advanced NSCLC. Although final results of most studies are needed to clarify the effect of this treatment in the PD-L1 low expression subgroup of patients, as well as possible differences between different ICPIs, current data suggest a possible more prominent OS advantage with the addition of anti-PD-1 pembrolizumab to chemotherapy, irrespective of the PD-L1 level and histology.

## Author Contributions

All authors listed have made a substantial, direct and intellectual contribution to the work, and approved it for publication.

### Conflict of Interest Statement

AA Merk Sharp & Dohme—honoraria for consulting (advisory board).

- Boerhinger Ingelheim—honoraria for consulting (advisory board) and research grant
- MSD—honoraria for consulting (advisory board)
- Roche—honoraria for consulting (advisory board)
- Astrazeneca-honoraria for consulting (advisory board)

GB: Merk Sharp & Dohme—honoraria for consulting (advisory board)

- Boerhinger Ingelheim—honoraria for consulting (advisory board)
- Janssen Cilag—honoraria for consulting (advisory board)
- Roche—honoraria for consulting (advisory board)

MD: honoraria and had roles as consultant or advisor for AstraZeneca, Lilly Pharma, Bristol Myers Squibb, MSD, Roche, Janssen, and Astellas.

The remaining author declares that the research was conducted in the absence of any commercial or financial relationships that could be construed as a potential conflict of interest.
