# Supplementary material for: Chemotherapy in Combination With Immune Checkpoint Inhibitors for the First-Line Treatment of Patients With Advanced Non-small Cell Lung Cancer: A Systematic Review and Literature-Based Meta-Analysis
Source: Front Oncol. 2019 Apr 16;9:264. doi: 10.3389/fonc.2019.00264 (PMC6478036; doi:10.3389/fonc.2019.00264)
Supplement: Supplementary file 1 [file Data_Sheet_1.PDF]

Figure 1. Eligible studies

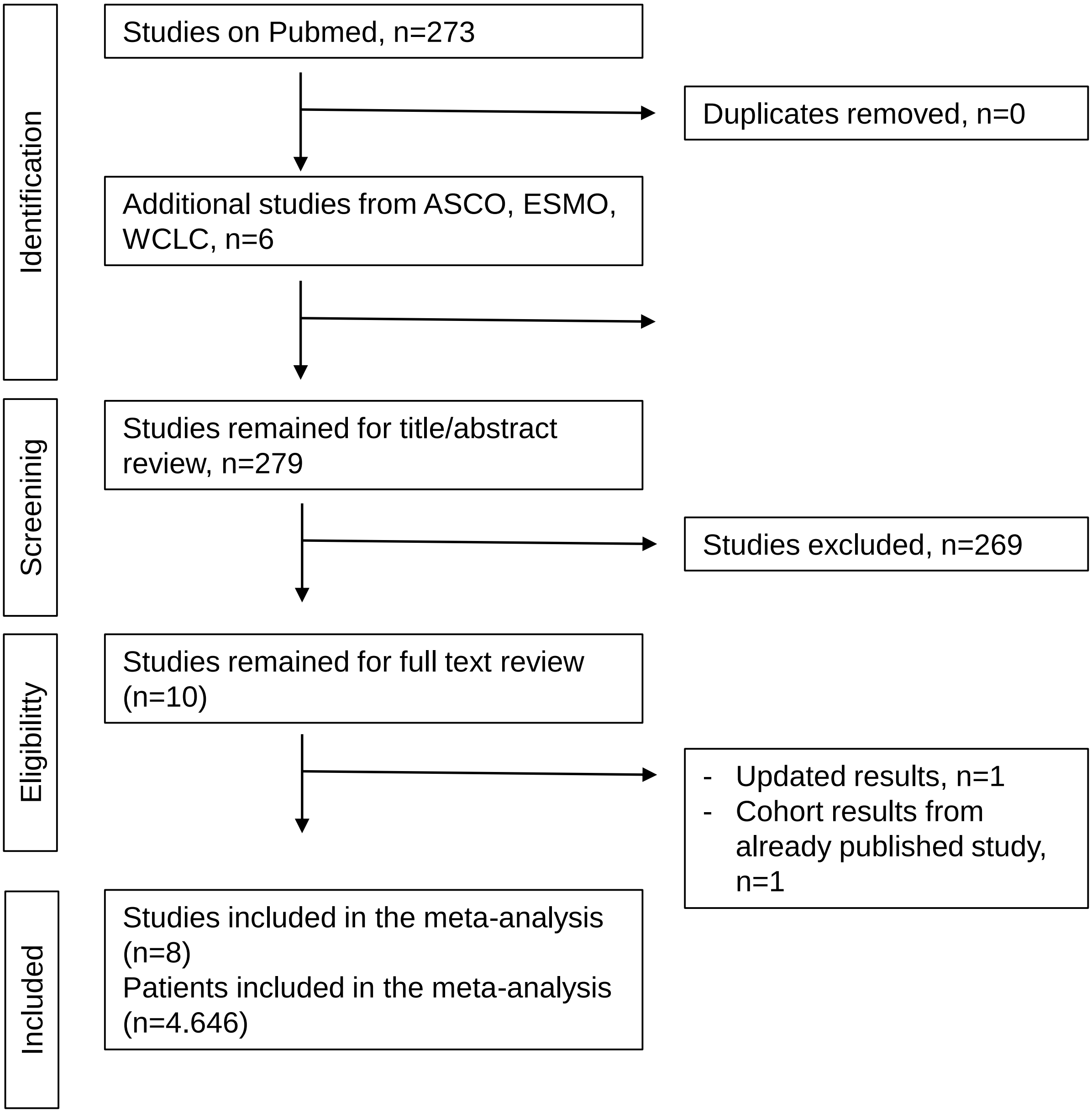

ASCO, American Society of Clinical Oncology; ESMO, European Society of Medical Oncology; WCLC, World Conference on Lung Cance.
